# CRISPR as a Diagnostic Tool

**DOI:** 10.3390/biom11081162

**Published:** 2021-08-06

**Authors:** Seohyun Kim, Sangmin Ji, Hye Ran Koh

**Affiliations:** Department of Chemistry, Chung-Ang University, Seoul 06974, Korea; tjgus474@cau.ac.kr (S.K.); say4125@cau.ac.kr (S.J.)

**Keywords:** CRISPR-Cas, diagnosis, gene detection, microRNA, single nucleotide polymorphism, DNA methylation, aptamer

## Abstract

Clustered regularly interspaced short palindromic repeats (CRISPR)-Cas system has recently gained growing attention as a diagnostic tool due to its capability of specific gene targeting. It consists of Cas enzymes and a guide RNA (gRNA) that can cleave the target DNA or RNA based on the sequence of the gRNA, making it an attractive genetic engineering technique. In addition to the target-specific binding and cleavage, the trans-cleavage activity was reported for some Cas proteins, including Cas12a and Cas13a, which is to cleave the surrounding single-stranded DNA or RNA upon the target binding of Cas-gRNA complex. All these activities of the CRISPR-Cas system are based on its target-specific binding, making it applied to develop diagnostic methods by detecting the disease-related gene as well as microRNAs and the genetic variations such as single nucleotide polymorphism and DNA methylation. Moreover, it can be applied to detect the non-nucleic acids target such as proteins. In this review, we cover the various CRISPR-based diagnostic methods by focusing on the activity of the CRISPR-Cas system and the form of the target. The CRISPR-based diagnostic methods without target amplification are also introduced briefly.

## 1. Introduction

**Figure 1:**
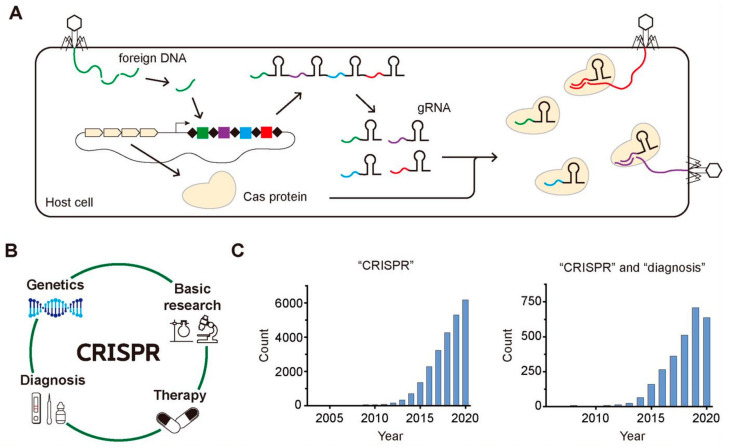
CRISPR-Cas system in the adaptive immune process of archaea or bacteria, its various application, and the growing attention. (**A**) The archaea and bacteria recognize the foreign DNA of invaders and insert the fragments of the foreign DNA into their CRISPR array. When the invaders attack the host cell again, their foreign DNAs are cleaved by Cas complexes with gRNA possessing the complementary sequences with the foreign DNAs. (**B**) The wide application of CRISPR-Cas system to various fields including basic research, genetics, therapy, and diagnosis. (**C**) The increasing number of published research papers per year that are searched by PubMed using one keyword of “CRISPR” for the left histogram and using two keywords of “CRISPR” and “diagnosis” for the right one.

Clustered regularly interspaced short palindromic repeats (CRISPR) is an array of short repeated DNA sequences interspaced with spacers having unique sequences, which has been found in approximately 50% of the bacterial genomes and in 87% of the genomes of archaea so far [1]. It was firstly discovered in the *Escherichia coli* genome in 1987 as repeated 29-nt fragments separated by 32-nt fragments, and the first evidence to show that the CRISPR-Cas system is a part of an adaptive immune system in archaea or bacteria was reported in 2005 [2,3]. As shown in Figure 1A, the host cell incorporates the pieces of the foreign gene of the invader in its own genome upon the first attack of the invader, memorizing the genetic information of foreign species. Then, it transcribes the small pieces of RNA containing the gene information of the foreign species known as guide RNA (gRNA) and translates CRISPR-associated (Cas) protein having endonuclease activity, producing Cas-gRNA complexes that can cleave the same foreign DNA of the invaders upon their second attack by using the genetic information stored in gRNA.

Soon after the several initial research about the function of the CRISPR-Cas system, it has been widely applied as a genome-editing tool because of its target specificity as well as the easiness to target any gene of interest just by changing the sequence of gRNA [4]. With the target-specific binding and cleavage of the CRISPR-Cas system, it has been applied to various areas such as genetics, other basic research, gene therapy, and molecular diagnosis (Figure 1B). Among various application of CRISPR-Cas system, we focus on its application in the diagnosis because lots of progress have been reported in this field recently. In addition, the widespread transmission of SARS-Cov-2 worldwide since last year accelerated the need to develop a rapid and accurate diagnostic tool [5]. The growing attention to CRISPR and CRISPR-based diagnostics are well reflected in the histograms displaying the number of the published research papers per year over the last few decades that were searched by PubMed (https://pubmed.ncbi.nlm.nih.gov, accessed on 10 May 2021) using one keyword, “CRISPR” (Figure 1C, left) and two keywords “CRISPR” and “diagnosis” (Figure 1C, right). The increasing number of papers implies the rapidly growing attention to CRISPR and CRISPR-based diagnosis.

In this review, we introduce the various diagnostic methods based on the CRISPR-Cas system, particularly focusing on the functional activity of Cas proteins used for detecting disease-related genetic molecules and the various form of the genetic molecules such as genomic DNA, mRNA, microRNA (miRNA), single nucleotide polymorphism (SNP) or DNA methylation. The majority of CRISPR-based diagnostic methods are based on detecting amplified nucleic acids, but the methods to detect non-nucleic acids target or to detect target genes without DNA amplification have been recently reported. CRISPR-based diagnostic methods for detecting non-nucleic acids and non-amplified targets are also discussed briefly in the last part of this review.

## 2. Gene Detection Based on the Various Activity of CRISPR-Cas

**Figure 2:**
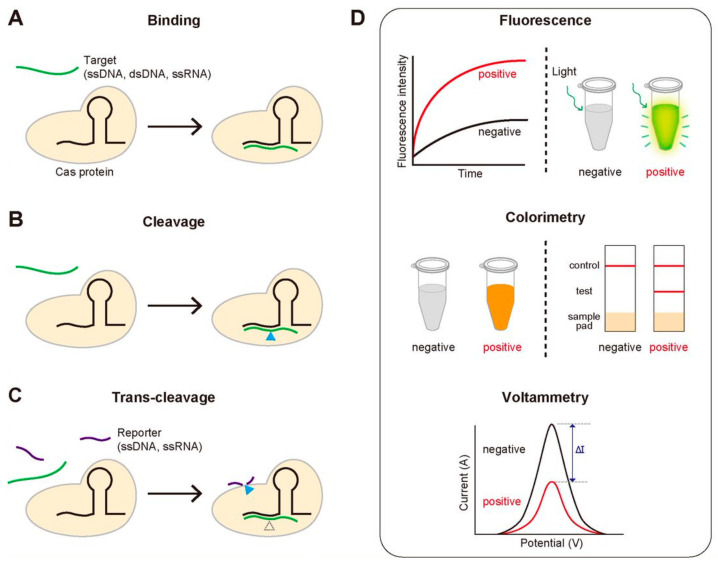
The key functional activities of the CRISPR-Cas system used for diagnosis and three routinely used signal detection methods. (**A**–**C**) The three activities of the CRISPR-Cas system for detecting target genes. (**A**) Sequence-specific target binding. Catalytically inactive Cas proteins bind to the target gene that is complementary to gRNA. (**B**) Sequence-specific target cleavage. Cas proteins cleave the target gene, followed by the sequence-specific binding. (**C**) Target-specific trans-cleavage. Some Cas proteins such as Cas12a or Cas13a non-specifically cleave the ssDNA or ssRNA nearby upon binding to the target gene. (**D**) Three widely-used signal detection techniques. The fluorescence, colorimetric or electric signal can be monitored to detect the existence of the target gene.

We note the key functional activities of the CRISPR-Cas system that are routinely employed in the diagnostic methods: (1) the sequence-specific binding to target DNA or RNA, (2) the sequence-specific cleavage of target DNA or RNA, and (3) the trans-cleavage of ssDNA or ssRNA that is activated by the sequence-specific binding or cleavage (Figure 2A–C). For the sequence-specific binding, most Cas proteins require a specific protospacer adjacent motif (PAM), a short DNA sequence that locates next to the target region. It is noteworthy that the first two activities are common for all the Cas proteins, but the last one is not (Table 1). Most diagnostic methods based on the CRISPR-Cas system use one of these three properties. Detection of disease-associated nucleic acids by using the CRISPR-Cas system can be achieved by using various detection platforms such as fluorescence, colorimetry, and voltammetry, contributing to generate the variety of the CRISPR-based diagnostic methods (Figure 2D). We summarize the good-to-know properties of widely-used Cas proteins for developing CRISPR-Cas-based diagnostic methods in Table 1.

### 2.1. Gene Detection Based on the Sequence-Specific Binding of CRISPR-Cas

Cas proteins bind to target DNA or RNA in a sequence-specific manner, which allows searching for the target gene of our interest simply just by changing the sequence of gRNA without any protein engineering or additional optimization of the experimental condition. Even though the main function of the CRISPR-Cas system is the cleavage of the target gene accompanied by the sequence-specific target search, some diagnostic methods based on the CRISPR-Cas system use the sequence-specific binding characteristics only without relying on the following cleavage activity (Figure 2A) [6,7,8]. These methods mostly use the deactivated Cas9 (dCas9) proteins where the dsDNA cleavage activity is eliminated by changing two amino acids in each cleavage-responsible domain of the Cas9 proteins, RuvC and HNH, displaying the strong binding affinity to dsDNA [9,10].

Guk et al. reported the CRISPR-mediated DNA fluorescence in situ hybridization (FISH) method for the detection of methicillin-resistant *staphylococcus aureus* (MRSA) [6]. In this assay, they could isolate the mecA gene in MRSA using a magnet by taking advantage of the target-specific binding of the dCas9-gRNA complex that was linked to the Ni-NTA magnetic bead. Then, the isolated DNAs were stained with a DNA intercalation dye, SYBR Green I, resulting in the fluorescence generation only in the case of the existence of MRSA. Zhang, Qian, and Wei et al. detected the gene of *Mycobacterium tuberculosis* by inducing the binding of two dCas9 proteins nearby in the existence of the target sequence [7]. They prepared two kinds of dCas9 proteins, one fused with the N-terminal part of the split luciferase and the other with the C-terminal of the one. In the existence of the DNA target of the interest, the two kinds of dCas9 proteins can bind close to each other, assembling the full construct of luciferase that emits light, so they could detect the gene of interest by measuring the enhancement of luminescence signal. They could successfully detect the 16S rRNA of *Mycobacterium tuberculosis* that was reversely transcribed to DNA and then amplified using conventional polymerase chain reaction (PCR), suggesting its potential as a diagnostic tool for *Mycobacterium tuberculosis*.

### 2.2. Gene Detection Based on the Sequence-Specific DNA Cleavage by CRISPR-Cas

The sequence-specific DNA cleavage activity of the CRISPR-Cas system makes it one of the most fascinating genetic engineering tools with an aim for the application to gene therapy because we can easily cleave out or modify the gene of interest. Even though CRISPR-Cas initially drew tremendous attention as a tool of gene therapy, some reported using the sequence-specific target cleavage by CRISPR-Cas even for developing a diagnostic method.

Quan, Langelier, and Kuchta et al. developed the finding low-abundance sequences by hybridization (FLASH) technique using the sequence-specific DNA cleavage by Cas9 [11]. With FLASH, they detected antimicrobial resistance sequences in *Staphylococcus* and drug resistance in the malaria parasite *Plasmodium falciparum* by sequencing followed by amplifying the gene of interest that was digested by Cas9 complex. In their assay, the cleaved DNA target by Cas9 can be amplified, not the other DNA, enriching the target gene only. Chen et al. detected circulating tumor DNA (ctDNA) using entropy-driven strand displacement reaction (ESDR), the enzyme-free amplification method, that was triggered by CRISPR-Cas9 [12]. They made three-dimensional structural graphene (3D GR)/AuPtPd nanoflower electrochemical biosensor coated with probes to capture the amplified DNA products in ESDR, which produces the change of electrochemical response according to the existence of the amplified products. In this assay, the site-specific DNA cleavage by CRISPR-Cas9 was the key factor to initiate the ESDR amplification of the target DNA that can be captured on 3D GR/AuPtPd nanoflower biosensor, making it possible to detect the low levels of ctDNA specifically.

### 2.3. Gene Detection Based on the Trans-Cleavage Activity of CRISPR-Cas

The sequence-specific binding to and cleavage of DNA or RNA are the main functions of the CRISPR-Cas system that make it one of the most attractive proteins in the field of genetics. However, the most successful diagnostic methods based on CRISPR so far are the ones to use its trans-cleavage activity, which was initially regarded as an unexpected artifact. Not all the Cas proteins possess the trans-cleavage activity; for instance, Cas9 does not, but Cas12a (also known as Cpf1) does (Table 1). After Cas12a binds and cleaves its target DNA, the generated triplex of Cas12a-gRNA-cleaved target DNA can fragmentize the surrounding ssDNA non-specifically, which is known as the trans-cleavage activity (Figure 2C). Unlike the target cleavage by the CRISPR-Cas system, the trans-cleavage of ssDNA or ssRNA is a multiple turn-over process [13], so the signal that relates with the specific target search can be amplified by the multiple processes of the trans-cleavage upon one-time target binding.

**Figure 3:**
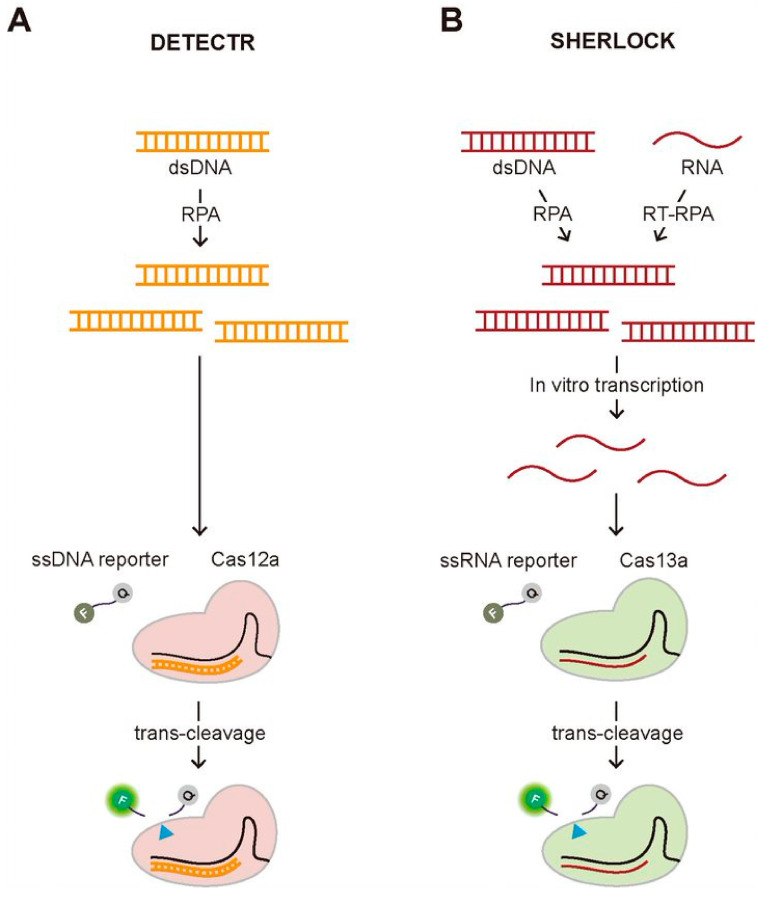
Two representative CRISPR-based diagnostic methods employing the trans-cleavage activity, DETECTR and SHERLOCK. (**A**) Schematic of DETECTR. The Cas12a-gRNA complex recognizes the target DNA that is amplified by RPA, an isothermal alternative to PCR employing a recombinase, a single-stranded DNA-binding protein, and a strand-displacing polymerase. Upon the target recognition, it fragmentizes the surrounding FQ-labeled ssDNA reporters, recovering the fluorescence. (**B**) Schematic of SHERLOCK. The Cas13a-gRNA complex binds to the target RNA that is in vitro transcribed from the amplified DNA by RPA or RT-RPA, which activates the trans-cleavage of the Cas13a-gRNA complex, cleaving the surrounding FQ-labeled ssRNA reporters.

One of the most well-known diagnostic methods using the trans-cleavage activity of the CRISPR system is Cas12a-assisted nucleic acid detection named as DNA endonuclease-targeted CRISPR trans reporter (DETECTR) (Figure 3A) [13]. In this assay, they employed a fluorophore quencher (FQ)-labeled reporter to monitor the trans-cleavage activity that was activated by the binding of the Cas12a-gRNA complex to the guide-complementary target DNA. In the existence of the target DNA, the trans-cleavage activity of the Cas12a-gRNA complex can be activated, resulting in the cleavage of surrounding FQ-labeled ssDNA reporters, which recovers the fluorescence of the FQ-labeled reporter by the release of the quencher. However, this assay requires the target amplification method, recombinase polymerase amplification (RPA), to achieve attomolar sensitivity for DNA detection. Most recently, the DETECTR assay was applied to detect SARS-Cov-2 causing Coronavirus disease 19 (COVID-19), validating its strength as a rapid and accurate detection assay for nucleic acids [14,15,16,17]. Soon after the first report to use the trans-cleavage activity of Cas12a for developing a DNA detection method, similar approaches have been reported using the different Cas proteins having the trans-cleavage activity such as Cas14a and Cascade-Cas3 [18,19]. Similar to Cas12a, Cas14a is a RNA-guided DNA endonuclease having trans-cleavage activity, but it is exceptionally compact, less than a half in size comparing to the typical Cas12a, and does not require PAM for target DNA recognition and cleavage [18]. Due to its non-specific DNase activity activated by the target recognition, Cas14-DETECTR was also developed, demonstrating the higher target specificity than Cas12a. More recently, Yoshimi et al. reported the trans-cleavage activity of Cascade-Cas3, the effector protein of Class 1 Type CRISPR-Cas system, and its application to detect SARS-Cov-2 [19]. The approach of their method, named Cas3-operated nucleic acid detection (CONAN), is similar to DETECTR, which combines and the cleavage of FQ-labeled reporter followed by target recognition with isothermal amplification methods. Unlike DETECTR, CONAN needs multiple Cas proteins that are characteristics of Class 1 CRISPR-Cas system, making it inconvenient to use, but the advantage over DETECTR is the higher specificity for single base-pair discrimination within the PAM site. Both DETECTR and CONAN employed the trans-cleavage effect of Cas proteins that were activated by target DNA-binding. Specific high-sensitivity enzymatic reporter unlocking (SHERLOCK) that was reported earlier than DETECTR also shares a similar approach combining the trans-cleavage activity of Cas proteins and isothermal amplification [20]. The main difference from DETECTR is to employ RNA-guided, RNA targeting CRISPR effector, Cas13a (also known as C2c2) protein that requires target RNA, not DNA, to activate the trans-cleavage activity. Therefore, in vitro transcription to generate target RNA followed by isothermal DNA amplification using either RPA or reverse transcriptase (RT)-RPA is necessary to activate the trans-cleavage activity of Cas13a, resulting in the cleavage of FQ-labeled ssRNA reporter (Figure 3B). Gootenberg et al. demonstrated that they could detect Zika and Dengue virus, pathogenic bacteria, and low-frequency cancer mutations in cell-free DNA (cfDNA) fragments using SHERLOCK with attomolar sensitivity [20]. Recently, SHERLOCK was also applied to detect SARS-CoV-2 [21,22,23].

## 3. Detection of miRNAs, SNPs, and DNA Methylation

### 3.1. miRNA Detection

A miRNA is a small single-stranded non-coding RNA having about 22 bps in length, which regulates gene expression by binding to target mRNAs. The gene regulation by miRNAs is critical for development, differentiation, growth, and metabolism, so the abnormal level of miRNAs was reported to result in various human diseases, including human cancers, cardiovascular diseases, and neuronal diseases [24]. For instance, several oncogenic miRNAs such as *miR-10b*, *miR-19b*, *miR-20a*, and *miR-21* were reported to relate to breast and brain cancer [25,26]. The *miR-129* and *miR-134* cause neuronal diseases such as Alzheimer’s disease and epilepsy [27,28]. Therefore, miRNAs have been actively investigated as a biomarker to diagnose the related various diseases, and numerous miRNA detection methods have been reported accordingly [29,30].

**Figure 4:**
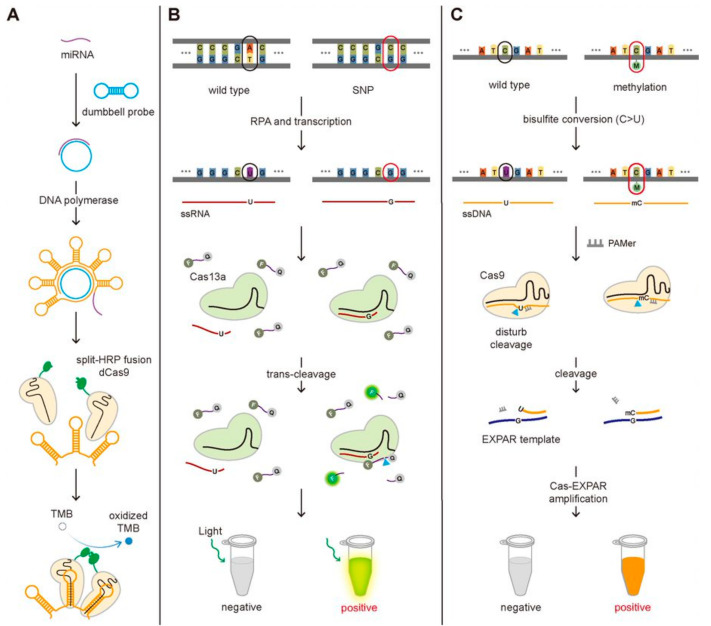
The CRISPR-Cas-based detection of the miRNA, SNP, and DNA methylation. (**A**) Schematic of the miRNA detection method. The binding of the miRNA to the dumbbell probe produces the repeated bundle of the hairpin structure where the dCas9 fused with split-HRP can bind. The miRNA induces the assembly of the split-HRP, which oxidizes TMB, changing the color of the solution. (**B**) Schematic of SNP detection method. With an optimal design of gRNA to differentiate the single-base mismatch in target RNA, SHERLOCK can be used to detect SNP. (**C**) Schematic of DNA methylation detection method. The cytosine residue in ssDNA, not methylated cytosine, can be converted to uracil by bisulfite conversion, generating the sequence difference at the methylation position between the two. The poor cleavage efficiency by Cas9 and lower binding affinity to EXPAR template for converted ssDNA (C > U) comparing with non-converted methylated ssDNA result in the different degree of Cas-EXPAR amplification, enabling to detect DNA methylation.

Even though the short length of miRNAs makes them more difficult to detect compared to the genomic DNA or mRNA, several studies have been reported to detect miRNAs employing the functional activity of the CRISPR-Cas system, the sequence-specific binding to target DNA/RNA and the accompanied trans-cleavage of surrounding ssDNA/ssRNA. Recently, the miRNA detection technique taking advantage of the sequence-specific binding of dCas9 was developed and applied to detect the miRNAs associated with non-small cell lung cancer (Figure 4A) [31]. They designed the dumbbell-shaped probe to be opened by binding with target miRNA, which can produce the repeated bundle structure by rolling circle amplification (RCA) [32]. The stem in the hairpin structure is specifically designed to bind the dCas9-gRNA complex that was fused with split-horseradish peroxidase (HRP)-C or -N. Close binding of dCas9-gRNA complexes can induce the assembly of HRP, and the combined HRP could oxidize tetramethylbenzidine (TMB), changing the color of the solution. In short, they could detect the target DNA by observing the color of the solution. Bruch and Baaske et al. successfully detected the *miR-19b* and *miR-20a* causing medulloblastoma by taking the benefit of the trans-cleavage of Cas13a activated by target RNA recognition [25,33]. In the existence of the miRNA, the Cas13a-gRNA-miRNA complex can fragmentize the 6-FAM-labeled ssRNA reporters, producing the electrochemical signal change [25]. In detail, only in the absence of the miRNA, the glucose oxidase (GOx) can be located close to the surface of the biosensor through 6-FAM with the treatment of the GOx-labeled antibody against 6-FAM, and then the GOx can catalyze its substrate, glucose, producing H_2_O_2_, that is amperometrically detected. The amperometric signal is proportional to the concentration of 6-FAM, which is inversely proportional to the amount of the miRNAs. In such a way, the existence of *miR-19b* and *miR-20a* were detected by monitoring the electrochemical signal change at the level of 2.2 nM.

### 3.2. SNP Detection

The SNPs are the most common type of genetic variation among people where a single base-pair (A, T, C, or G) variation occurs in every 1000 nucleotides on average. Most SNPs do not affect health or development, but some are responsible for a particular disease or drug response [34]. The SNPs found to be associated with disease have been widely used for diagnostic purposes [35]. In addition, the SNPs are not the unique feature of human beings, but they are found in whole organisms, from viruses and bacteria to mammals. SNPs in the disease-causing pathogens such as bacteria or viruses provide information of pathogens such as endemicity, transmission mode, and clinical outcomes, making it a useful biomarker for the diagnosis and the appropriate treatments [36]. For example, detection of SNPs in the hepatitis B virus (HBV), the most common viral infection in humans, tells the genotypes of the HBV, B or C, where genotype C is associated with more active disease and higher risk of liver cirrhosis comparing with genotype B [37,38].

CRISPR-based SNPs detection methods have been reported for the diagnosis of certain diseases. Ke et al. employed SHERLOCK to detect SNP (C347G and C369T) in HBV to genotype the virus and Gootenberg et al. to detect SNP (T2573G) in epidermal growth factor receptor (EGFR) gene known to associate with lung cancer (Figure 4B) [20]. This method needs the Cas13a protein and gRNA that contains the complementary sequence to the HBV virus or the EGFR mutation sequence. After the RPA amplification and the transcription of the amplified target DNA, the Cas13a-gRNA complexes are added together with the FQ-labeled ssRNA probes. Only the target RNA possessing a single-point mutation can bind to the Cas13a-gRNA complex activating its trans-cleavage activity for the surrounding ssRNA because it possesses the perfect complementarity with gRNA, not the wild-type DNA. In this way, the SNPs (C347G and C369T in HBV and T2573G in EGFR) were detected by monitoring the increase in the fluorescence signal caused by the cleavage of FQ-labeled ssRNA at the level of attomolar [20,37]. Harrington et al. detected the SNP of the HERC2 gene that decides the eye color (brown or blue eye). They developed Cas14-DETECTR where Cas14a substitutes Cas12a in DETECTR assay and applied to detect SNPs in HERC2 gene by taking advantage of Cas14a that is more sensitive to the sequence mismatch between gRNA and target DNA in the internal region (9–14 nt) than Cas12a [18]. Pardee et al. developed a CRISPR-based technique to identify the strain of the Zika virus by combining isothermal RNA amplification, toehold switch RNA sensors, and CRISPR-Cas9 module. Among two huge strains of Zika viruses, American and African, African Zika virus is different from American in having two SNPs, T7240A and G7264A, in comparison with American Zika, so the detection of SNPs is required for the strain identification. They could differentiate these two strains by designing gRNA to locate the SNP site at the PAM region where American Zika has the appropriate PAM sequence but not African, making American Zika, not African, cleaved by Cas9. The uncleaved dsDNA (African Zika) was transcribed to RNA that contains the trigger sequence for toehold reaction but not the cleaved one (American Zika), enabling to identify the strain of Zika virus [39].

### 3.3. DNA Methylation Detection

DNA methylation is an epigenetic process where methyl groups are added to DNA, primarily at cytosine residues in both prokaryotes and eukaryotes [40]. DNA methylation regulates gene expression, typically reducing transcription by affecting the interaction of DNA with transcription factors or chromatin proteins. Therefore, the aberrant DNA methylation results in various diseases, which have been widely observed in many cancer types, autoimmune diseases, metabolic disorders, psychological disorders, and aging [41,42]. This makes disease-specific DNA methylation a potential biomarker for various diseases, for instance, septin 9 (SEPT9)-methylated DNA for colorectal cancer screening [43].

A CRISPR-Cas9 triggered exponential amplification reaction method (CAS-EXPAR) was developed by combining the allele-specific cleavage of Cas9 and the rapid amplification of EXPAR and applied to detect the DNA methylation [44]. They treated the target DNA with sodium bisulfite that converts non-methylated cytosine into uracil, generating a single-base mutation at the methylated position between the target DNA associated with PAM-presenting oligonucleotide (PAMer) and the guide RNA. Because the only methylated DNA not the DNA with non-methylated cytosine contains the perfect complementarity with the gRNA, it is cleaved by the Cas9-gRNA complex much better than non-methylated DNA and then associated better with the EXPAR template for the amplification. The better cleavage efficiency by Cas9 and higher binding affinity to EXPAR template for methylated DNA comparing with the non-methylated DNA result in the different degrees of Cas-EXPAR amplification. In this scheme, the methylated DNA can be amplified through the EXPAR, which was quantified by measuring the fluorescence signal from the fluorescent dye to stain dsDNA (Figure 4C).

## 4. Non-nucleic Acid Target Detection

The CRISPR-Cas system recognizes the nucleic acids, either DNA or RNA, in a sequence-specific manner, allowing the easy application to detect the nucleic acids associated with diseases. In some cases, the non-nucleic acids such as proteins, adenosine 5′-triphosphates (ATP), and telomerase activities need to be quantified because they are also correlated with certain diseases [45,46,47]. The CRISPR-Cas system has also been employed to detect non-nucleic acids even though its main function originates from the sequence-specific binding to nucleic acids. For developing CRISPR-Cas to detect non-nucleic acids, the aptamer has been frequently employed, which is an oligonucleotide or peptide to bind to a specific target molecule such as proteins or even small molecules such as ATP [48,49,50,51].

**Figure 5:**
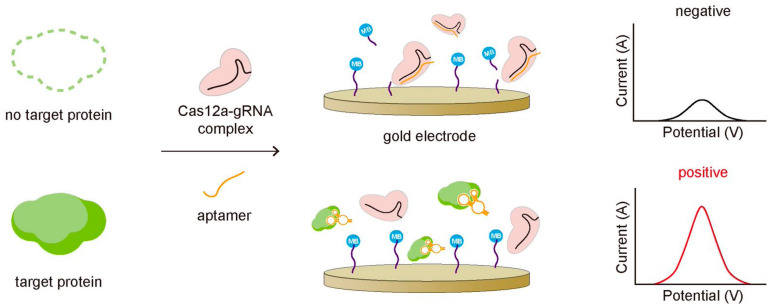
Proteins can be detected by combining CRISPR-Cas with the protein-specific nucleic acid aptamer. The aptamer is designed to bind stronger to target protein than to the CRISPR-Cas system, so it cannot activate the trans-cleavage of the CRISPR-Cas system when the target protein exists, producing a strong amperometric signal due to the intact MB close to the gold electrode.

Dai et al. reported detecting transforming growth factor β1 (TGF-β1), a biomarker for hepatocellular carcinoma, by employing an aptamer that binds to both Cas12a-gRNA and TGF-β1 [49]. In the absence of the target protein, the aptamer bound to the Cas12a-gRNA complex activates the trans-cleavage activity of Cas12a, cleaving the methylene blue (MB)-labeled ssDNA reporters coated on the gold electrode surface and making it released from the surface (Figure 5). The release of MB resulted in the decrease in current on the electrode, which can be monitored to quantify TGF-β1 protein. In the presence of TGF-β1, on the other hand, the aptamer binds to TGF-β1 rather than Cas12a-gRNA complex preventing the activation of the Cas12a-gRNA complex for its trans-cleavage activity. In this assay, the stronger binding affinity of the aptamer to target protein was essential. In the other case, Zhao, Zhang, and Qiu et al. detected the transmembrane protein, CD63, in an exosome based on the CRISPR-Cas system [50]. The exosomes are related to various cancers, so detection of exosomes can be used to diagnose exosome-associated diseases [52]. In order to detect exosomes, they used the CD63-specific aptamer partially bound with its blocker, which cannot bind to the Cas12a-gRNA complex because of the blocker. The blocker was designed to be removed by CD63 binding due to the stronger binding affinity of the aptamer to CD63 than the blocker. In the absence of exosome, the aptamer remained bound with the blocker cannot activate the trans-cleavage activity of the Cas12a-gRNA complex, leaving FQ-labeled ssDNA reporters intact without changing the fluorescent signal. In the presence of exosome, however, the aptamer binding to the CD63 protein in the exosome can release the blocker, allowing the aptamer to bind the Cas12a-gRNA complex. Then, it activated trans-cleavage of FQ-labeled ssDNA reporters, recovering the fluorescent signal. In this way, they successfully quantified the exosome at the level of 3 × 10^3^ particles per microliter by monitoring the change of the fluorescence signal.

## 5. Non-Amplification Methods

Most CRISPR-based diagnostic methods to detect the nucleic acids are combined with target amplification typically by PCR, making them not to avoid the intrinsic drawbacks of PCR such as the additional time-consuming amplification procedure and the requirement of the expensive reagents and instruments. Moreover, the additional primer design and optimization step are often required to reduce the non-specific amplification. Therefore, the non-amplification diagnostic methods are highly in demand, and several non-amplification methods combining with the CRISPR-Cas system have been recently reported [25,53].

Hajian et al. detected Duchenne muscular dystrophy (DMD)-associated exon deletion by using graphene field-effect transistor (gFET) coated with the dCas9-gRNA complexes [53]. The electric current of gFET increases upon the binding of the target DNA to the dCas9-gRNA complex, and the high electric conductivity of graphene makes this method very sensitive to the binding of the target DNA even without the amplification step. They detected the existence of the exons 3 and 51 of the dystrophin gene, which are commonly deleted in DMD patients, by measuring the electric current in healthy people and DMD patients and confirmed the deletion of the exons 3 and 51 for DMD patients. Another CRISPR-based non-amplification method was reported by Bruch and Baaske et al., who detected *miR-19b*, a potential brain tumor marker [25]. They used the trans-cleavage activity of Cas13a with electrochemical signal readout as described earlier. In this assay, they achieved to detect *miR-19b* using gRNA that was designed to be complementary with *miR-19b* without any target amplification due to the highly sensitive electric signal. They showed the inverse correlation between the amperometric signal and the amount of the miRNAs and measured the low amperometric signal in the serum samples of children suffering from brain cancer, demonstrating the feasibility of their assay as a target amplification-free tool for detecting miRNAs.

## 6. Conclusions

CRISPR-Cas system has emerged as a fascinating and powerful diagnostic tool by taking advantage of the sequence-specific target binding and cleavage as well as the target-specific trans-cleavage. The trans-cleavage activity makes them more attractive because it occurs multiple times for one target binding by the CRISPR-Cas complex, enhancing the detection sensitivity. Nucleic acids are the main target of the CRISPR-Cas system, so various forms of nucleic acids such as genomic DNA, mRNA, miRNA, SNP, and methylated DNA that relate to diseases have been successfully detected and quantified by combining the CRISPR-Cas system with target amplification. Moreover, several recent approaches have been reported to be capable of detecting non-nucleic acids or free of target amplification even though the majority of CRISPR-based diagnostics targets nucleic acids that need to be amplified using various amplification techniques, including PCR, RPA, EXPAR, and so on. The easily programmable character of CRISPR-Cas makes it to detect any gene of interest, allowing to develop several diagnostic tools for SARS-CoV-2 as well. The diagnostic tool based on the CRISPR-Cas system could be the next-generation standard due to its programmability and capability of searching for the target sequence quickly and specifically even in the genomic DNA.

## Figures and Tables

**Table 1:** The characteristics of various Cas proteins.

Cas Protein	Class	Target	PAM	Trans-Cleavage
Cas3	Class 1	dsDNA	AAG	Yes (ssDNA)
Cas9	Class 2	dsDNA	NGG	No
Cas12a	Class 2	dsDNA/ssDNA	TTTN	Yes (ssDNA)
Cas13a	Class 2	ssRNA	*	Yes (ssRNA)
Cas14a	Class 2	ssDNA	-	Yes (ssDNA)

* Some Cas13a orthologues require a PAM-like sequence motif named as protospacer flanking site.

## Data Availability

Not applicable.

## Abbreviations

CRISPR	Clustered Regularly Interspaced Short Palindromic Repeats
gRNA	guide RNA
Cas	CRISPR-associated
miRNA	microRNA
SNP	Single Nucleotide Polymorphism
dCas9	deactivated Cas9
FISH	Fluorescence In Situ Hybridization
MRSA	Methicillin-Resistant *Staphylococcus Aureus*
PCR	Polymerase Chain Reaction
FLASH	Finding Low Abundance Sequences by Hybridization
ctDNA	circulating tumor DNA
ESDR	Entropy-driven Strand Displacement Reaction
3D GR	three-Dimensional structural GRaphene
DETECTR	DNA Endonuclease-Targeted CRISPR Trans Reporter
FQ	Fluorophore Quencher
RPA	Recombinase Polymerase Amplification
PAM	Protospacer Adjacent Motif
CONAN	Cas3-Operated Nucleic Acid Detection
SHERLOCK	Specific High-sensitivity Enzymatic Reporter unLOCKing
RT-RPA	Reverse Transcriptase Recombinase Polymerase Amplification
cfDNA	cell-free DNA
RCA	Rolling Circle Amplification
HRP	Horseradish peroxidase
TMB	Tetramethylbenzidine
GOx	Glucose Oxidase
HBV	Hepatitis B Virus
EGFR	Epidermal Growth Factor Receptor
SEPT9	SEPTin 9
CAS-EXPAR	CRISPR-Cas9 triggered EXPonential Amplification Reaction
TGF-β1	Transforming Growth Factor β1
MB	Methylene Blue
DMD	Duchenne Muscular Dystrophy
gFET	graphene Field-Effect Transistor

## References

[1] Sorek R., Kunin V., Hugenholtz P. (2008). CRISPR—A widespread system that provides acquired resistance against phages in bacteria and archaea. Nat. Rev. Microbiol..

[2] Mojica F.J.M., Díez-Villaseñor C.S., García-Martínez J., Soria E. (2005). Intervening Sequences of Regularly Spaced Prokaryotic Repeats Derive from Foreign Genetic Elements. J. Mol. Evol..

[3] Ishino Y., Shinagawa H., Makino K., Amemura M., Nakata A. (1987). Nucleotide sequence of the iap gene, responsible for alkaline phosphatase isozyme conversion in Escherichia coli, and identification of the gene product. J. Bacteriol..

[4] Mali P., Yang L., Esvelt K.M., Aach J., Guell M., DiCarlo J.E., Norville J.E., Church G.M. (2013). RNA-guided human genome engineering via Cas9. Science.

[5] Udugama B., Kadhiresan P., Kozlowski H.N., Malekjahani A., Osborne M., Li V.Y.C., Chen H., Mubareka S., Gubbay J.B., Chan W.C.W. (2020). Diagnosing COVID-19: The Disease and Tools for Detection. ACS Nano.

[6] Guk K., Keem J.O., Hwang S.G., Kim H., Kang T., Lim E.-K., Jung J. (2017). A facile, rapid and sensitive detection of MRSA using a CRISPR-mediated DNA FISH method, antibody-like dCas9/sgRNA complex. Biosens. Bioelectron..

[7] Zhang Y., Qian L., Wei W., Wang Y., Wang B., Lin P., Liu W., Xu L., Li X., Liu D. (2017). Paired Design of dCas9 as a Systematic Platform for the Detection of Featured Nucleic Acid Sequences in Pathogenic Strains. ACS Synth. Biol..

[8] Moon J., Kwon H.J., Yong D., Lee I.C., Kim H., Kang H., Lim E.K., Lee K.S., Jung J., Park H.G. (2020). Colorimetric Detection of SARS-CoV-2 and Drug-Resistant pH1N1 Using CRISPR/dCas9. ACS Sens..

[9] Jinek M., Chylinski K., Fonfara I., Hauer M., Doudna J.A., Charpentier E. (2012). A Programmable Dual-RNA–Guided DNA Endonuclease in Adaptive Bacterial Immunity. Science.

[10] Doudna J.A., Charpentier E. (2014). The new frontier of genome engineering with CRISPR-Cas9. Science.

[11] Quan J., Langelier C., Kuchta A., Batson J., Teyssier N., Lyden A., Caldera S., McGeever A., Dimitrov B., King R. (2019). FLASH: A next-generation CRISPR diagnostic for multiplexed detection of antimicrobial resistance sequences. Nucleic Acids Res..

[12] Chen M., Wu D., Tu S., Yang C., Chen D., Xu Y. (2021). CRISPR/Cas9 cleavage triggered ESDR for circulating tumor DNA detection based on a 3D graphene/AuPtPd nanoflower biosensor. Biosens. Bioelectron..

[13] Chen J.S., Ma E., Harrington L.B., Da Costa M., Tian X., Palefsky J.M., Doudna J.A. (2018). CRISPR-Cas12a target binding unleashes indiscriminate single-stranded DNase activity. Science.

[14] Ding X., Yin K., Li Z., Lalla R.V., Ballesteros E., Sfeir M.M., Liu C. (2020). Ultrasensitive and visual detection of SARS-CoV-2 using all-in-one dual CRISPR-Cas12a assay. Nat. Commun..

[15] Pang B., Xu J., Liu Y., Peng H., Feng W., Cao Y., Wu J., Xiao H., Pabbaraju K., Tipples G. (2020). Isothermal Amplification and Ambient Visualization in a Single Tube for the Detection of SARS-CoV-2 Using Loop-Mediated Amplification and CRISPR Technology. Anal. Chem..

[16] Ma P., Meng Q., Sun B., Zhao B., Dang L., Zhong M., Liu S., Xu H., Mei H., Liu J. (2020). MeCas12a, a Highly Sensitive and Specific System for COVID-19 Detection. Adv. Sci..

[17] Broughton J.P., Deng X., Yu G., Fasching C.L., Servellita V., Singh J., Miao X., Streithorst J.A., Granados A., Sotomayor-Gonzalez A. (2020). CRISPR–Cas12-based detection of SARS-CoV-2. Nat. Biotechnol..

[18] Harrington L.B., Burstein D., Chen J.S., Paez-Espino D., Ma E., Witte I.P., Cofsky J.C., Kyrpides N.C., Banfield J.F., Doudna J.A. (2018). Programmed DNA destruction by miniature CRISPR-Cas14 enzymes. Science.

[19] Yoshimi K., Takeshita K., Yamayoshi S., Shibumura S., Yamauchi Y., Yamamoto M., Yotsuyanagi H., Kawaoka Y., Mashimo T. (2020). Rapid and accurate detection of novel coronavirus SARS-CoV-2 using CRISPR-Cas3. medRxiv.

[20] Gootenberg J.S., Abudayyeh O.O., Lee J.W., Essletzbichler P., Dy A.J., Joung J., Verdine V., Donghia N., Daringer N.M., Freije C.A. (2017). Nucleic acid detection with CRISPR-Cas13a/C2c2. Science.

[21] Hou T., Zeng W., Yang M., Chen W., Ren L., Ai J., Wu J., Liao Y., Gou X., Li Y. (2020). Development and evaluation of a rapid CRISPR-based diagnostic for COVID-19. PLoS Pathog..

[22] Joung J., Ladha A., Saito M., Kim N.-G., Woolley A.E., Segel M., Barretto R.P.J., Ranu A., Macrae R.K., Faure G. (2020). Detection of SARS-CoV-2 with SHERLOCK One-Pot Testing. N. Engl. J. Med..

[23] Patchsung M., Jantarug K., Pattama A., Aphicho K., Suraritdechachai S., Meesawat P., Sappakhaw K., Leelahakorn N., Ruenkam T., Wongsatit T. (2020). Clinical validation of a Cas13-based assay for the detection of SARS-CoV-2 RNA. Nat. Biomed. Eng..

[24] Das J., Podder S., Ghosh T.C. (2014). Insights into the miRNA regulations in human disease genes. BMC Genet..

[25] Bruch R., Baaske J., Chatelle C., Meirich M., Madlener S., Weber W., Dincer C., Urban G.A. (2019). CRISPR/Cas13a-Powered Electrochemical Microfluidic Biosensor for Nucleic Acid Amplification-Free miRNA Diagnostics. Adv. Mater..

[26] Iorio M.V., Ferracin M., Liu C.-G., Veronese A., Spizzo R., Sabbioni S., Magri E., Pedriali M., Fabbri M., Campiglio M. (2005). MicroRNA Gene Expression Deregulation in Human Breast Cancer. Cancer Res..

[27] Hosseinian S., Arefian E., Rakhsh-Khorshid H., Eivani M., Rezayof A., Pezeshk H., Marashi S.-A. (2020). A meta-analysis of gene expression data highlights synaptic dysfunction in the hippocampus of brains with Alzheimer’s disease. Sci. Rep..

[28] Brodie M.J., Barry S.J.E., Bamagous G.A., Norrie J.D., Kwan P. (2012). Patterns of treatment response in newly diagnosed epilepsy. Neurology.

[29] Tian T., Wang J., Zhou X. (2015). A review: microRNA detection methods. Org. Biomol. Chem..

[30] Raoof R., Bauer S., El Naggar H., Connolly N.M.C., Brennan G.P., Brindley E., Hill T., McArdle H., Spain E., Forster R.J. (2018). Dual-center, dual-platform microRNA profiling identifies potential plasma biomarkers of adult temporal lobe epilepsy. EBioMedicine.

[31] Qiu X.Y., Zhu L.Y., Zhu C.S., Ma J.X., Hou T., Wu X.M., Xie S.S., Min L., Tan D.A., Zhang D.Y. (2018). Highly Effective and Low-Cost MicroRNA Detection with CRISPR-Cas9. ACS Synth. Biol..

[32] Deng R., Tang L., Tian Q., Wang Y., Lin L., Li J. (2014). Toehold-initiated Rolling Circle Amplification for Visualizing Individual MicroRNAs In Situ in Single Cells. Angew. Chem. Int. Ed..

[33] Murphy B.L., Obad S., Bihannic L., Ayrault O., Zindy F., Kauppinen S., Roussel M.F. (2013). Silencing of the miR-17∼92 Cluster Family Inhibits Medulloblastoma Progression. Cancer Res..

[34] Risch N.J. (2000). Searching for genetic determinants in the new millennium. Nature.

[35] Gray I.C., Campbell D.A., Spurr N.K. (2000). Single nucleotide polymorphisms as tools in human genetics. Hum. Mol. Genet..

[36] Srinivas P.R., Kramer B.S., Srivastava S. (2001). Trends in biomarker Res. for cancer detection. Lancet Oncol..

[37] Ke Y., Huang S., Ghalandari B., Li S., Warden A.R., Dang J., Kang L., Zhang Y., Wang Y., Sun Y. (2021). Hairpin-Spacer crRNA-Enhanced CRISPR/Cas13a System Promotes the Specificity of Single Nucleotide Polymorphism (SNP) Identification. Adv. Sci..

[38] Chan H.L.Y., Wong M.L., Hui A.Y., Hung L.C.T., Chan F.K.L., Sung J.J.Y. (2003). Hepatitis B virus genotype C takes a more aggressive disease course than hepatitis B virus genotype B in hepatitis B e antigen-positive patients. J. Clin. Microbiol..

[39] Pardee K., Green A.A., Takahashi M.K., Braff D., Lambert G., Lee J.W., Ferrante T., Ma D., Donghia N., Fan M. (2016). Rapid, Low-Cost Detection of Zika Virus Using Programmable Biomolecular Components. Cell.

[40] Moore L.D., Le T., Fan G. (2013). DNA Methylation and Its Basic Function. Neuropsychopharmacology.

[41] Fernandez A.F., Assenov Y., Martin-Subero J.I., Balint B., Siebert R., Taniguchi H., Yamamoto H., Hidalgo M., Tan A.-C., Galm O. (2012). A DNA methylation fingerprint of 1628 human samples. Genome Res.

[42] Jin Z., Liu Y. (2018). DNA methylation in human diseases. Genes Dis..

[43] Warren J.D., Xiong W., Bunker A.M., Vaughn C.P., Furtado L.V., Roberts W.L., Fang J.C., Samowitz W.S., Heichman K.A. (2011). Septin 9 methylated DNA is a sensitive and specific blood test for colorectal cancer. BMC Med..

[44] Huang M., Zhou X., Wang H., Xing D. (2018). Clustered Regularly Interspaced Short Palindromic Repeats/Cas9 Triggered Isothermal Amplification for Site-Specific Nucleic Acid Detection. Anal. Chem..

[45] Xu X., Zheng L., Yuan Q., Zhen G., Crane J.L., Zhou X., Cao X. (2018). Transforming growth factor-β in stem cells and tissue homeostasis. Bone Res..

[46] Coulouarn C., Factor V.M., Thorgeirsson S.S. (2008). Transforming growth factor-beta gene expression signature in mouse hepatocytes predicts clinical outcome in human cancer. Hepatology.

[47] Shay J.W., Bacchetti S. (1997). A survey of telomerase activity in human cancer. Eur. J. Cancer.

[48] Cheng M., Xiong E., Tian T., Zhu D., Ju H.-q., Zhou X. (2021). A CRISPR-driven colorimetric code platform for highly accurate telomerase activity assay. Biosens. Bioelectron..

[49] Dai Y., Somoza R.A., Wang L., Welter J.F., Li Y., Caplan A.I., Liu C.C. (2019). Exploring the Trans-Cleavage Activity of CRISPR-Cas12a (cpf1) for the Development of a Universal Electrochemical Biosensor. Angew. Chem. Int. Ed..

[50] Zhao X., Zhang W., Qiu X., Mei Q., Luo Y., Fu W. (2020). Rapid and sensitive exosome detection with CRISPR/Cas12a. Anal. Bioanal. Chem..

[51] Xiong Y., Zhang J., Yang Z., Mou Q., Ma Y., Xiong Y., Lu Y. (2020). Functional DNA Regulated CRISPR-Cas12a Sens. for Point-of-Care Diagnostics of Non-Nucleic-Acid Targets. J. Am. Chem. Soc..

[52] Xu J., Liao K., Zhou W. (2018). Exosomes Regulate the Transformation of Cancer Cells in Cancer Stem Cell Homeostasis. Stem Cells Int..

[53] Hajian R., Balderston S., Tran T., deBoer T., Etienne J., Sandhu M., Wauford N.A., Chung J.-Y., Nokes J., Athaiya M. (2019). Detection of unamplified target genes via CRISPR-Cas9 immobilized on a graphene field-effect transistor. Nat. Biomed. Eng..

